# Emotional Intelligence and Creative Performance in the Information and Communication Technology (ICT) Sector: The Mediating Role of Psychological Well-Being and the Moderating Role of Cultural Intelligence

**DOI:** 10.3390/jintelligence14090214

**Published:** 2026-09-05

**Authors:** Ibrahim Yikilmaz, Lutfi Surucu, Mustafa Bekmezci, Bulent Cetinkaya, Kemal Erogluer

**Affiliations:** 1 Department of Management and Organization, Faculty of Business Administration, Kocaeli University, Kocaeli 41380, Türkiye; 2 Department of Business Administration, Faculty of Business and Economics, Altinbas Cyprus University, Mersin 10, Nicosia 99010, Türkiye; lutfi.surucu@wpu.edu.tr; 3 Department of Defence Studies, Military Academy, National Defence University, Ankara 06420, Türkiye; mustafa.bekmezci@msu.edu.tr; 4 Department of Business Administration, Faculty of Business, European Leadership University, Mersin 10, Famagusta 99500, Türkiye; bulent@elu.edu.tr; 5 Department of Business Administration, Faculty of Humanities and Social Sciences, Atlas University, Istanbul 34408, Türkiye; kemal.erogluer@atlas.edu.tr

**Keywords:** emotional intelligence, creative performance, psychological well-being, cultural intelligence, information and communication technology sector

## Abstract

Emotional intelligence is the capacity to understand, regulate, and manage one’s emotions, and it has significant effects on employees’ psychological adjustment, interpersonal relationships, and job performance. However, the specific psychological mechanisms by which emotional intelligence influences employees’ creative performance, and the conditions under which this relationship is strengthened, remain not fully understood. The aim of this study is to examine the mediating role of psychological well-being and the moderating role of cultural intelligence in the relationship between emotional intelligence and creative performance among individuals working in the information and communication technology (ICT) sector. Research data were obtained from 409 participants working in large-scale information and communication technology companies operating in Turkey, and the research hypotheses were tested using the bootstrap method with the Hayes PROCESS Macro. The findings show that emotional intelligence is positively related to psychological well-being and creative performance. Furthermore, psychological well-being significantly mediates the relationship between emotional intelligence and creative performance. The results show that cultural intelligence strengthens this indirect effect, and the contribution of emotional intelligence to creative performance is more pronounced at higher levels of cultural intelligence. The findings indicate that emotional intelligence is associated with creative performance both directly and indirectly through psychological well-being. However, the strength of this indirect association appears to vary across employees’ levels of cultural intelligence, which is considered an individual capability rather than a characteristic of the work environment. In this respect, the study contributes to the literature by examining psychological well-being as an explanatory pathway and cultural intelligence as an individual-level boundary condition in the association between emotional intelligence and creative performance.

## 1. Introduction

Emotional intelligence (EI) has attracted considerable scholarly attention over the last two decades as an important personal resource that helps individuals navigate complex social and professional environments. Beyond the ability to perceive, understand, and regulate emotions, emotional intelligence has been associated with a wide range of positive outcomes, including psychological adjustment, interpersonal effectiveness, well-being, and individual performance (Mayer et al., 2004; Martins et al., 2010; Sánchez-Álvarez et al., 2016). As contemporary workplaces become increasingly dynamic, technology-driven, and interaction-intensive, understanding how emotional intelligence contributes to positive employee outcomes has become an important issue for both researchers and practitioners.

Although emotional intelligence has been linked to numerous beneficial outcomes, the processes that may help explain these relationships remain less clear. Recent evidence suggests that the relationships between emotional intelligence and employee outcomes may not be adequately explained by simple direct associations and may involve different psychological processes and individual-level conditions (Sánchez-Álvarez et al., 2016; dos Santos et al., 2025). In particular, emotional intelligence has been associated with creativity and innovative behavior, yet the psychological processes that may help explain this relationship remain insufficiently understood (Xu et al., 2019). Therefore, examining how emotional intelligence is related to creative performance and identifying the psychological pathway and individual-level boundary condition involved in this relationship remain important areas for further investigation.

Among the various outcomes associated with emotional intelligence, creative performance has attracted increasing scholarly attention due to its strategic importance in contemporary organizations. Creativity enables employees to generate novel and useful ideas, adapt to changing work demands, and contribute to organizational innovation. Consequently, understanding the emotional and psychological resources that foster creative performance has become an increasingly important research objective.

Although creativity has long been considered within the framework of cognitive capacity, intelligence level, and individual talent, recent studies have shown that it cannot be explained solely by analytical thinking or technical expertise; it is also closely related to an individual’s emotional, psychological, and social processes and capital (Amabile et al., 2005; Baas et al., 2008; Yu et al., 2019). Recent findings in the organizational behavior and positive psychology literature indicate that factors such as an employee’s emotional experiences, psychological well-being, and interpersonal adaptability can significantly affect their creative capacity (Fredrickson, 2001; Isen, 1999, Acar et al., 2021). This approach has moved beyond the traditional view of creativity as a cognitive output, leading to a shift toward more holistic models that examine how employees’ psychological resources influence creative performance (Rego et al., 2012). In this context, emotional intelligence—the capacity to understand, regulate, and appropriately manage one’s own emotions and those of others—emerges as a crucial individual resource for explaining creative performance and for overcoming creative tasks that involve uncertainty, risk of failure, and cognitive pressure (Mayer et al., 2004; Darvishmotevali et al., 2018; Xu et al., 2019). While a study that provided detailed analyses of the research revealed a significant and positive relationship between emotional intelligence and creativity (Xu et al., 2019), recent research indicates that this relationship may not always manifest directly or with equal strength. Studies, particularly those in high-tech sectors, suggest that the relationship between emotional intelligence and creative performance may involve various psychological and organizational processes (dos Santos et al., 2025), highlighting the need to examine the psychological factors that may help explain this relationship. Accordingly, recent research has increasingly called for process-oriented approaches that examine not only whether emotional intelligence is associated with adaptive and performance-related outcomes, but also the pathways and conditions involved in these relationships.

In recent years, holistic and process-based approaches have come to the forefront in shaping and explaining employee behavior. Within this scope, psychological well-being has been considered a mediator in the relationship between emotional intelligence and creative performance. Psychological well-being refers to positive evaluations of one’s life, psychological balance, and positive functioning (Ryff & Keyes, 1995). Positive psychological states have also been associated with greater cognitive flexibility and creativity (Baas et al., 2008; Acar et al., 2021). According to Fredrickson’s (2001) Broaden and Build Theory, positive psychological states expand individuals’ intellectual repertoire and support more flexible cognitive processes. Indeed, meta-analytic and empirical studies examining the relationship between positive psychological states and creativity also support this approach (Baas et al., 2008; Rego et al., 2012).

From this perspective, emotional intelligence can be viewed as a personal resource associated with positive psychological functioning, while psychological well-being may represent an explanatory pathway linking this resource to desirable work outcomes. Accordingly, the Broaden-and-Build Theory provides a useful framework for understanding why emotional resources may be associated with creativity through positive psychological functioning.

The individual resources employees possess may not yield the same results across all conditions and environments. Especially in work environments where cultural diversity, remote work systems, and multidisciplinary team structures are prevalent, such as the information technology sector, contextual factors are increasingly decisive for organizational and individual outcomes (Russo et al., 2021). In addition, information technology employees frequently work in project-based environments characterized by cross-functional collaboration, rapid technological change, tight deadlines, and continuous knowledge exchange. Such conditions increase the importance of emotional and cultural capabilities that enable employees to adapt, collaborate, and generate innovative solutions. Indeed, employees in the IT sector come from diverse areas of expertise and interact within different communication norms and international team structures. Therefore, it can be said that not only the capacity of employees to manage emotional processes but also their ability to adapt to different cultural contexts becomes important. At this point, cultural intelligence, as the individual’s capacity to function effectively and adapt to different cultural norms, stands out as an important individual resource (Ang et al., 2007). Although studies in the literature show that cultural intelligence is related to psychological adjustment, interpersonal interaction, and stress management (Bücker et al., 2016; Rockstuhl & Van Dyne, 2018), studies on how cultural intelligence plays a role in the process between emotional intelligence and creative performance are quite limited. Based on the existing literature, three important research gaps can be identified. First, although emotional intelligence has been associated with employee creativity, the psychological processes that may help explain this relationship remain insufficiently understood (Darvishmotevali et al., 2018; dos Santos et al., 2025). Darvishmotevali et al. (2018) examined environmental uncertainty as a mediating mechanism and cultural intelligence as a moderator of the relationship between emotional intelligence and creative performance among frontline hotel employees. Their study provided important evidence that this relationship may depend on individual and environmental conditions. However, it did not examine employees’ psychological well-being as an explanatory pathway. This distinction is important because environmental uncertainty reflects employees’ perceptions of their work environment, whereas psychological well-being concerns their positive psychological functioning. Second, prior research suggests that emotional intelligence is associated with employee outcomes through different psychological processes and positive psychological resources (Ye et al., 2024). Nevertheless, limited attention has been paid to whether psychological well-being statistically explains part of the relationship between emotional intelligence and creative performance. Third, although cultural intelligence has been linked to adaptation and interpersonal effectiveness, less is known about whether this individual capability strengthens the association between emotional intelligence and psychological well-being and, consequently, the indirect association with creative performance. Furthermore, recent discussions in the emotional intelligence literature emphasize the need to move beyond direct-effect models and examine the explanatory pathways and boundary conditions involved in the relationship between emotional intelligence and individual functioning (Mayer et al., 2004; Sánchez-Álvarez et al., 2016). In line with these calls, examining psychological well-being as an explanatory pathway and cultural intelligence as an individual-level boundary condition may provide a more comprehensive understanding of the relationship between emotional intelligence and creative performance in contemporary work environments. Such an approach helps clarify both the psychological pathway involved in this relationship and the conditions under which the indirect association may be stronger or weaker (Xu et al., 2019; dos Santos et al., 2025).

Therefore, this study aims to investigate the relationship between emotional intelligence and creative performance in individuals working in the information technology sector. This research examines the mediating role of psychological well-being and the moderating role of cultural intelligence together. The study differs from previous research by focusing on psychological well-being, rather than environmental uncertainty, as the explanatory pathway and by examining whether cultural intelligence conditions the relationship between emotional intelligence and psychological well-being. In this way, the proposed model seeks to clarify whether emotional intelligence is associated with creative performance partly through employees’ positive psychological functioning and whether this indirect association varies across levels of cultural intelligence. The study therefore offers a context-specific examination of how emotional, psychological, and cultural capabilities are jointly associated with creative performance.

## 2. Literature Review and Hypothesis Development

### 2.1. Emotional Intelligence and Creative Performance

Creative performance involves developing new, original, and applicable ideas within work processes that meet the expectations of internal and external customers or addressing existing problems from a different perspective and proposing new solutions. In information- and technology-intensive industries, beyond technical expertise, individual resources such as collaboration, teamwork, emotional regulation, adaptability, and psychological resilience contribute more significantly. Indeed, in the face of intense time pressure and complex task structures, an employee’s technical or analytical thinking skills alone are insufficient; they also need the capacity to manage emotional processes both individually and as part of a team. In this context, emotional intelligence is defined as an individual’s capacity to understand, regulate, and appropriately use their own emotions and those of others (Mayer et al., 2004). In creative tasks, when negative pressures such as uncertainty and failure are combined with time and cost pressures, managing emotions becomes particularly important in tasks involving high cognitive demands. Indeed, an emotionally competent employee can effectively manage stressful conditions; they can better mitigate the suppressive effects of negative emotions and thoughts on success, becoming more open to alternative solutions and collaboration (Mayer et al., 2004). Furthermore, studies have shown a positive correlation between emotional intelligence and creative performance (Darvishmotevali et al., 2018; Xu et al., 2019; Stawicki et al., 2023).

Studies in technology-intensive organizations and sectors also reveal significant findings indicating that the impact of emotional intelligence on creative performance involves a mechanism far more complex than a one-way relationship can explain. A study of employees in high-tech firms showed that many variables, including role stressors, self-efficacy, and role conflict—variables related to the workplace, individual capacity, and task—as well as team structure and high-performance expectations, can have an impact (dos Santos et al., 2025). This finding suggests that evaluating the impact of emotional intelligence on creative performance through mechanisms such as psychological well-being may be more meaningful.

From a theoretical perspective, the relationship between emotional intelligence and creative performance can be explained within the framework of the Broaden-and-Build Theory (Fredrickson, 2001). According to this theory, positive emotions broaden individuals’ intellectual output and behavioral repertoire, enabling them to approach events more flexibly, develop different perspectives on processes, and generate new solutions. Conversely, negative emotions such as fear, anxiety, stress, and fear of failure narrow an individual’s attention and redirect their cognitive resources to managing the current threat. Given that creativity requires high cognitive flexibility, the ability to develop alternative thinking and approaches, and the capacity to integrate diverse information and resources, how an employee manages their emotional processes is critically important to creative performance. At this point, individuals with high emotional intelligence are competent in accurately identifying their own and others’ emotions and developing their behavior accordingly (Mayer et al., 2004). The ability to control one’s own emotions and accurately assess others’ emotions helps maintain an employee’s psychological balance, even under challenging conditions such as heavy workloads, time pressure, role conflicts, interpersonal disagreements, and performance expectations. Being emotionally balanced allows individuals to view problems or events encountered within the natural dynamics of social interaction not as threats, but as opportunities for learning and growth, enabling them to develop new ideas and creative solutions. Therefore, emotional intelligence should be considered not only an individual competency that enables the management of emotions, but also a strategic personal resource that contributes to the preservation of cognitive and psychological resources supporting the emergence of creativity. The fact that emotionally balanced employees are more open to different perspectives, can evaluate problems in alternative ways, and can generate new ideas despite negative conditions provides an important theoretical basis for explaining the relationship between emotional intelligence and creative performance. Indeed, research in software engineering indicates that emotional intelligence is relevant to managing interpersonal relationships, sustaining team productivity, and setting and maintaining team goals when employees handle changing requirements (Madampe et al., 2024). Similarly, modern software development processes require employees to use both technical and interpersonal skills, and emotional intelligence is a significant resource that supports team collaboration and performance (Araújo et al., 2025). Furthermore, the technostress, uncertainty, and intense communication requirements that accompany digital work environments further increase the importance of employees’ emotional regulation skills.

Individuals working in the information technology sector often operate within team-based, project-oriented work structures. In these settings, employees need to demonstrate not only technical expertise but also strong communication, stress management, and adaptability skills. In software development, digital services, and technology-based business processes, where creative problem-solving is crucial, employees’ ability to maintain emotional balance, approach negative feedback constructively, and remain open to different solutions can support creative performance. Based on these arguments, the following hypothesis is proposed:

**H1.**  *Emotional intelligence is positively associated with creative performance.*

### 2.2. The Mediating Role of Psychological Well-Being

Although the number of studies examining the impact of emotional intelligence on creative performance is increasing, the specific psychological mechanisms underlying this relationship remain poorly understood. Investigating these mechanisms, particularly in information- and technology-intensive work environments under constant pressure for change and with complex team dynamics, constitutes an important area of research.

Within the positive psychological approach, which emphasizes the importance of individuals’ positive psychological evaluations for cognitive flexibility, creative thinking, and problem-solving (Acar et al., 2021), studies increasingly examine resources such as psychological well-being to explain creative performance. According to Fredrickson’s (2001) Broaden and Build Theory, positive psychological states expand individuals’ intellectual repertoires and support more flexible cognitive processes. Within this approach, psychological well-being is considered an important mechanism that enables individuals to transform their personal resources into creative behaviors.

Empirical studies examining the relationship between emotional intelligence and psychological well-being support the strength of this interaction. A meta-analytic study emphasizes a significant association between emotional intelligence and subjective well-being, highlighting that emotion-regulation skills significantly affect individuals’ positive psychological functioning and experiences (Sánchez-Álvarez et al., 2016). Another meta-analysis highlights the supportive role of emotional intelligence in psychological health and in stress-management adjustment processes (Martins et al., 2010), suggesting that psychological well-being influenced by emotional intelligence may be an important individual resource.

In technology and information-intensive sectors, this relationship is even more critical. Employees face psychological risks such as high cognitive load, time pressure, constantly changing task expectations, project-based work structures, hybrid or remote work models, social isolation, and digital burnout (Wei et al., 2022; Wells et al., 2023; Singh et al., 2022). Therefore, psychological well-being is important not only for individual health but also for organizational performance. Employees with high emotional intelligence can maintain their psychological well-being and balance by managing emotions in stressful situations, regulating negative emotions in a controlled manner, and functioning more effectively in interpersonal relationships.

On the other hand, studies and meta-analyses on psychological well-being and creative performance emphasize that positive affect and organizational dynamics have a significant effect on creativity, increasing cognitive flexibility and making individuals more open to generating new ideas (Baas et al., 2008; Amabile et al., 2005; Alimoğlu Özkan et al., 2022; Graziotin et al., 2014). A recent study of employees in information and technology-intensive industries shows that the effect of emotional intelligence on creativity is influenced by indirect psychological and organizational variables (psychological processes such as role stressors and self-efficacy) (dos Santos et al., 2025). Therefore, this mechanism can be explained by psychological mechanisms such as psychological well-being.

Taken together, these findings suggest that emotional intelligence may contribute to employees’ creative performance not only directly but also indirectly through positive psychological processes. Employees with higher emotional intelligence are more likely to maintain psychological well-being by effectively regulating emotions and coping with work-related challenges. In turn, higher levels of psychological well-being may facilitate cognitive flexibility, openness to new experiences, and creative problem-solving. Therefore, psychological well-being may represent an important mechanism through which emotional intelligence is translated into creative performance. Accordingly, the following hypothesis is proposed to be tested:

**H2.**  *Psychological well-being mediates the relationship between emotional intelligence and creative performance.*

### 2.3. The Moderating Role of Cultural Intelligence

Cultural intelligence is a competence that enables an individual to function effectively in different cultural contexts to adapt to different social norms, understand different perspectives, and behave more flexibly in cross-cultural interactions (Ang et al., 2007). Especially in sectors with high diversity and project-based work, such as information technology, the need to interact closely with team members from different areas of expertise makes cultural intelligence even more important for employee experiences.

The extant literature contains several studies demonstrating a close relationship between cultural intelligence and psychological adjustment processes. Cultural intelligence facilitates individuals’ social and psychological adaptation capacity, positively influencing their stress management, communication quality, and interpersonal effectiveness levels (Bücker et al., 2016; Rockstuhl & Van Dyne, 2018). These effects indicate that cultural intelligence can emerge as an important individual resource that supports employees’ psychological functioning. However, it appears that cultural intelligence is related not only to adaptation processes but also to employees’ creative and innovative behaviors. Indeed, Hu et al. (2019) showed that employees with high levels of cultural intelligence exhibit higher creative performance. Similarly, Yang et al. (2024) demonstrated that cultural intelligence supports employee creativity and contributes to individuals’ ability to evaluate different perspectives more effectively.

Furthermore, Fan et al. (2020) reported that cultural intelligence can positively influence employees’ innovative behaviors by promoting knowledge sharing. The capacity to understand diverse perspectives, draw on a range of information sources, and adapt to different working styles is a key component of cultural intelligence. High intelligence levels can facilitate the development of new ideas and the generation of alternative solutions in individuals. Therefore, cultural intelligence is considered an important individual resource that supports creative performance, especially in highly diverse work environments.

The joint consideration of emotional intelligence, psychological well-being, creative performance, and cultural intelligence is central to the present study. Emotional intelligence concerns individuals’ capacity to understand and manage emotions, whereas cultural intelligence reflects their capability to function effectively in culturally diverse situations (Ang et al., 2007). Accordingly, cultural intelligence is conceptualized in this study as an individual capability, not as a characteristic of the organizational or cultural environment.

Cultural intelligence is expected to moderate the relationship between emotional intelligence and psychological well-being. Employees with higher cultural intelligence may interpret unfamiliar social cues more accurately, adapt their behavior to different interaction norms, and experience fewer difficulties in culturally diverse encounters (Ang et al., 2007; Rockstuhl & Van Dyne, 2018). These capabilities may help them use their emotional skills more effectively when managing interpersonal demands. Therefore, the positive association between emotional intelligence and psychological well-being is expected to be stronger at higher levels of cultural intelligence. Because psychological well-being is, in turn, associated with creative performance, cultural intelligence may also condition the indirect association between emotional intelligence and creative performance through psychological well-being. Cultural intelligence is thus treated as an individual-level boundary condition of the proposed relationship rather than as evidence of the cultural characteristics of the participating organizations.

Accordingly, the following hypotheses are proposed to be tested:

**H3.**  *Cultural intelligence moderates the positive relationship between emotional intelligence and psychological well-being, such that this relationship is stronger at higher levels of cultural intelligence.*

**H4.**  *The positive indirect association between emotional intelligence and creative performance through psychological well-being is moderated by cultural intelligence, such that the indirect association is stronger at higher levels of cultural intelligence.*

**Figure 1:**
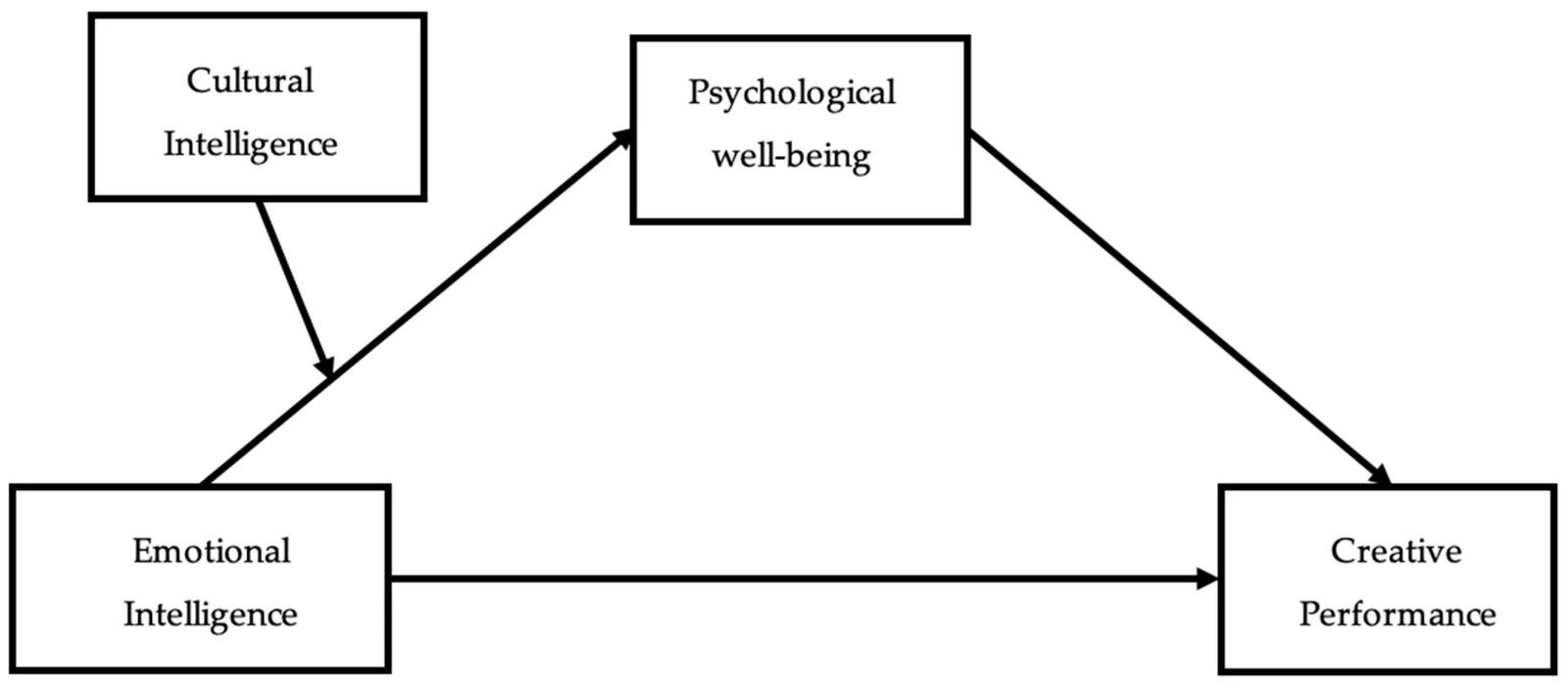
Research model.

The research model is presented below (Figure 1):

## 3. Methodology

### 3.1. Sample and Data Collection Procedures

The target population of the study consisted of employees working in large-scale ICT companies in Turkey. However, the accessible sample was limited to employees of two companies headquartered in Istanbul, Turkey, that agreed to participate in the study. The data collection process aimed to reach IT companies considered large-scale in terms of employee numbers and corporate structure. To this end, the human resources and senior management units of these companies were contacted, and official invitation letters explaining the purpose and scope of the research were sent. As a result of interviews with company officials who responded, three companies initially agreed to participate in the research; however, one of these companies withdrew during the process. Data were collected electronically via an e-survey form through internal company communication and messaging platforms, with the approval of company officials. Although a mass message was sent to employees, participation in the survey was entirely voluntary, and only employees who wished to participate did so. To minimize common methodological biases and participant concerns, an informed consent form was included on the first page of the survey. The form stated that participation was entirely voluntary, that the data would not be shared with company management, and that participants’ identities would remain anonymous. Although the study excluded participants’ nationality information, all participants had Turkish reading and comprehension skills because the survey was prepared in Turkish.

A total of 441 survey forms were collected at the end of the three-week data collection period (3–25 March 2026). Due to corporate confidentiality policies, the total number of employees was not disclosed by company management, and a net response rate could not be calculated. The researchers meticulously reviewed the survey data and excluded 32 surveys because they were considered incomplete or indicative of careless responding. Of these surveys, 11 showed all responses to the same option (e.g., all 1 or all 5), 7 showed responses forming systematic patterns (e.g., responses ordered from 5 to 1 and then from 1 to 5), and 14 showed that more than 50% of the questions remained unanswered. Final analyses were conducted on 409 valid survey responses (N = 409).

Regarding the demographic profile of the participants, 160 (39.1%) were female and 249 (60.9%) were male. In terms of marital status, 221 participants (54%) were married and 188 (46%) were single. In terms of age distribution, 70 participants (17.1%) were 25 years and under, 203 (49.6%) were between 26 and 35 years old, 89 (21.8%) were between 36 and 45 years old, and 47 (11.5%) were 45 years and over.

### 3.2. Measurement Tools

This study used measurement tools previously developed and used in various research studies. Regarding the Turkish forms of the scales, to our knowledge, no validated Turkish versions existed in the literature. Therefore, it was decided to adapt the original scales into Turkish using a systematic translation-back translation process. The translation-back translation approach suggested by Brislin (1970) was followed in adapting the scales for use in the Turkish context. In the first stage, two independent academics translated the scale items from English into Turkish. Next, two different academics independently retranslated the Turkish forms into English, without reference to the previous translations or the original English items. The forward and back-translation results were compared; necessary revisions were made until differences in meaning, ambiguities in expression, and conceptual inconsistencies were resolved. In the final stage, an academic expert in Turkish Language and Literature checked the items’ linguistic clarity and comprehensibility. Thus, the study considered not only word-level equivalence but also conceptual and semantic equivalence. A pilot study was conducted with 32 participants before the main sample. The pilot study aimed to evaluate item comprehensibility, expression clarity, and any ambiguity in participants’ interpretations. The pilot confirmed the comprehensibility of the materials, after which the main survey was administered. Psychological well-being was scored using a 7-point Likert scale, while other variables were measured using a 5-point Likert scale. Participants’ emotional intelligence was measured using 16 items developed by Law et al. (2004). An example item is “Quite capability to control my own emotions.” Psychological well-being was measured using three items developed by Baker and Kim (2020). An example item is “I am satisfied with achieving personal goals and hopes.” Cultural intelligence was measured using nine items developed by Darvishmotevali et al. (2018), drawing on the work of Konanahalli et al. (2014) and Ang et al. (2007). An example item is “I like interacting with individuals from different cultures.” Creative performance was measured using seven items developed by Mutonyi et al. (2020). An example item is “I often have new ideas to accomplish my work task.” The eight-item “brand trust” scale developed by Delgado-Ballester (2004) was used as a marker variable in the study. Consistent with previous research, demographic variables (gender, status and age) were controlled for in the analyses (Darvishmotevali et al., 2018).

### 3.3. Statistical Analysis

The data analysis process consisted of two main stages. In the first stage, descriptive statistics were reported, followed by the assessment of the constructs’ validity and reliability. In the second stage, the research hypotheses were tested using Hayes’ PROCESS Macro. The mediation analysis was followed by a first-stage moderated mediation analysis using PROCESS Model 7, in which cultural intelligence moderated the relationship between emotional intelligence and psychological well-being. PROCESS automatically generated the interaction term between emotional intelligence and cultural intelligence. The interaction term was estimated using the centering procedure implemented in PROCESS. Missing item-level responses were addressed using the mean assignment method before the main analyses. The analysis used 5000 bootstraps and 95% confidence intervals. Process Macro was chosen because it provides a robust empirical framework for testing mediating and moderating models using the bootstrap method and is frequently preferred in the literature (Hayes et al., 2017).

## 4. Results

### 4.1. Common Method Bias

To minimize the risk of common method bias (CMB) arising from the study’s cross-sectional design, procedural measures suggested by Podsakoff et al. (2003) were rigorously applied. In this context, to reduce participants’ social desirability concerns, it was emphasized that there were no true or false answers to the statements and that anonymity was guaranteed. Items were randomized to prevent artificial correlation between latent constructs. Furthermore, to ensure methodological diversity and disrupt participants’ response patterns, one scale in the study was designed with a 7-point Likert scale. In contrast, the other three scales were designed with a 5-point Likert scale. Finally, the questionnaire was kept short to reduce cognitive fatigue (Podsakoff et al., 2003).

In addition to procedural measures to mitigate the risk of common method bias (CMB), the presence of this risk in the data was also assessed using various statistical methods. First, the results of Harman’s one-factor test revealed that the first factor explained only 28.7% of the total variance (threshold < 50%), and therefore, no dominant factor was found in the data. To address the well-documented limitations of Harman’s test in the literature and to provide a more rigorous evaluation, a marker variable (MLMV) was included in the analysis (Lindell & Whitney, 2001). Accordingly, the 8-item “brand trust” scale developed by Delgado-Ballester (2004), which is conceptually unrelated to the research variables, was used as the marker variable, and the coefficients of the structural relationships were compared before and after including the marker variable in the model. Before the marker variable was included in the model, it was found that the effect of emotional intelligence on creative performance changed from B = 0.328 to B = 0.321 (2.1% change), the effect of emotional intelligence on psychological well-being changed from B = 0.182 to B = 0.179 (1.6% change), and the effect of psychological well-being on creative performance changed from B = 0.287 to B = 0.280 (2.4% change). These results show that including the marker variable in the model led to only limited changes in the magnitude of the basic structural relationships. In addition, the preserved direction and statistical significance of the relationships indicate that the marker variable did not significantly change the basic relationships in the research model. Additionally, Bagozzi et al.’s (1991) recommendations were followed, and it was determined that the correlations among the constructs remained below the recommended threshold of 0.90. Finally, the VIF (Variance Inflation Factor) values ranged from 1.7 to 2.9, all below the conservative threshold of 3.3 recommended by Podsakoff et al. (2003). Taken together, the findings provide no strong evidence that common methodological bias significantly affects the research findings.

### 4.2. Reliability and Validity Analyses

Before hypothesis testing, the validity of each construct in the model was evaluated using convergent and discriminant analyses, and reliability was assessed using Cronbach’s alpha (α) and composite reliability (CR). Reliability results are presented in Table 1.

The reliability of the constructs was assessed because all Cronbach’s alpha, CR and McDonald’s Omega estimates were above 0.7, as suggested by Hair et al. (2021). The findings in Table 1 show that the reliability values of the constructs are well above the suggested threshold.

In Table 1, the statistically significant and strongly loaded item loadings indicate that the items represent the relevant factor well (Sürücü et al., 2024). Average variance extracted (AVE) values were considered for convergent validity. AVE values exceeded 0.50 for all constructs. This confirms convergent validity as suggested by Fornell and Larcker (1981). The Fornell–Larcker criterion and the heterotrait–monotrait (HTMT) ratio were considered in assessing discriminant validity (Table 2).

For the Fornell and Larcker criterion, the square root of AVE was compared with the inter-construct correlations to determine whether they exceeded the AVE values. As shown in Table 2, the square roots of AVE are greater than the correlation values. This indicates that the constructs have discriminant validity. For a more comprehensive evaluation of discriminant validity, HTMT ratios, which yield more robust results according to the Fornell and Larcker criterion, were calculated. According to Henseler et al. (2015), discriminant validity can be considered when the HTMT ratios are below 0.85. The findings in Table 2 confirm that the constructs possess discriminant validity.

To evaluate the validity of the measurement model, a series of confirmatory factor analyses (CFAs) were performed using AMOS 25.0. The CFAs used maximum likelihood estimation. The Likert-type items were treated as continuous variables in the analyses because they used five- and seven-point response categories. The proposed four-factor research model was compared with various substitution models, and the results are presented in Table 3.

As shown in Table 3, the assumed four-factor research model has better fit indices than other models (χ^2^/df= 2.489; CFI = 0.977; GFI = 0.958; TLI = 0.949; NFI = 0.976 and RMSEA = 0.048).

### 4.3. Hypothesis Testing

After verifying the validity and reliability of the data collected in the study, the PROCESS Macro developed by Hayes (2017) was used to test the hypotheses empirically. The mediation model was examined first, followed by the first-stage moderated mediation model using PROCESS Model 7. To increase the reliability of parameter estimates, a 5000-resampling (bootstrap) procedure was applied, and the findings were evaluated at the 95% confidence level. The analysis results are reported in Table 4.

The findings show that emotional intelligence is positively associated with creative performance (B = 0.328, SE = 0.063, 95% CI [0.204, 0.450], *p* < .05) and with psychological well-being (B = 0.182, SE = 0.032, 95% CI [0.121, 0.245], *p* < .05). Furthermore, psychological well-being strongly predicts creative performance (B = 0.287, SE = 0.046, 95% CI [0.197, 0.378], *p* < .05). Thus, Hypothesis 1 is supported.

To test Hypothesis 2, the indirect effect of emotional intelligence on creative performance via psychological well-being was examined. The findings reveal that the indirect effect is significant (B = 0.053, SE = 0.025, 95% CI [0.009, 0.106], *p* < .05). Thus, Hypothesis 2 is supported.

**Figure 2:**
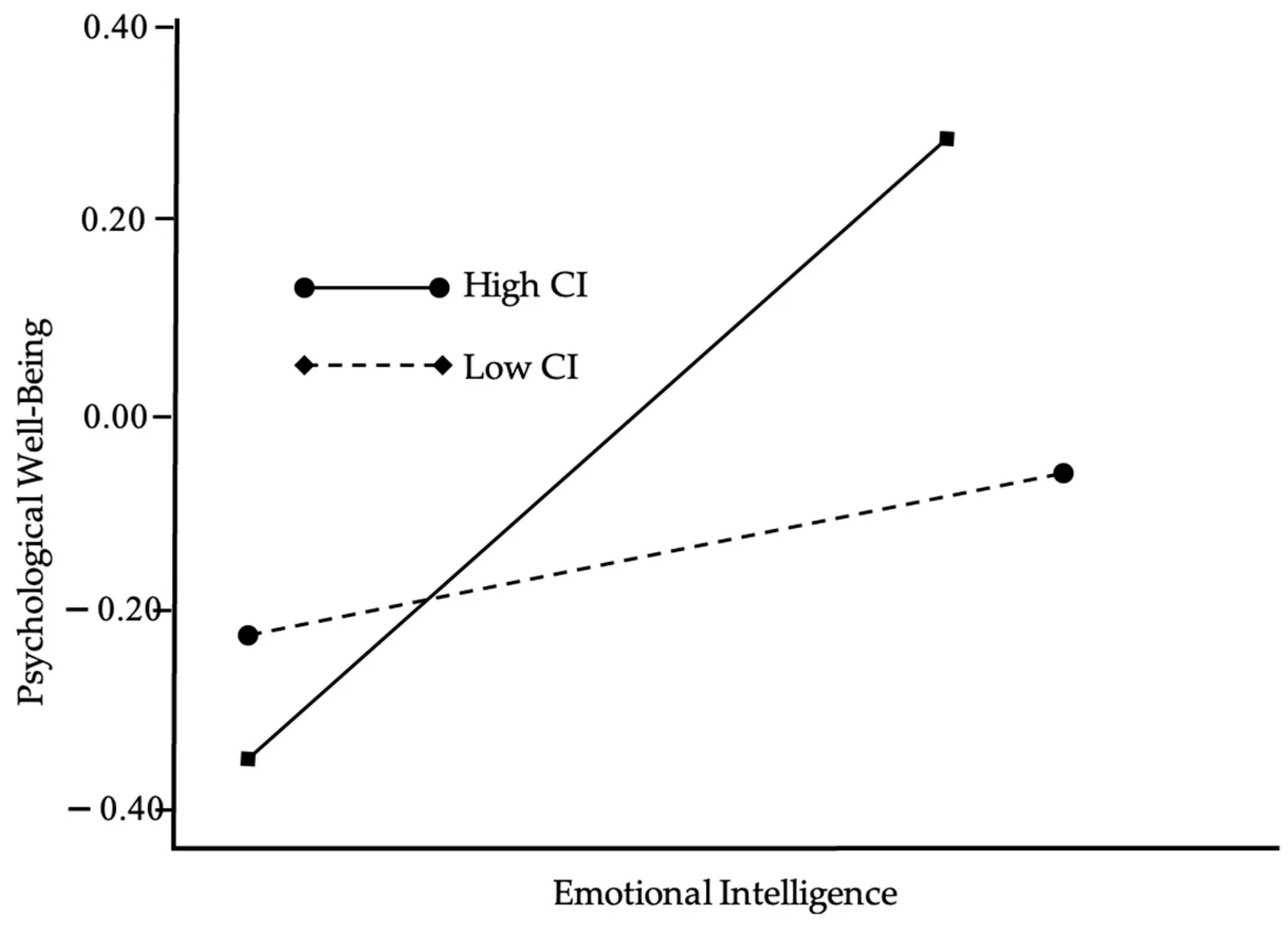
Regulatory Impact Graph.

Hypotheses H3 and H4 of the study suggest that the effect of emotional intelligence on creative performance through psychological well-being depends on the conditional role of cultural intelligence. To test this hypothesis, the significance of the moderated mediation index was assessed. The findings show that the moderated mediation index is significant, indicating that the effect of emotional intelligence on creative performance through psychological well-being depends on the conditional role of cultural intelligence (B = 0.075, SE = 0.030, 95% CI [0.016, 0.134], *p* < .05). A simple graph, created to visualize the effect, is presented in Figure 2.

The findings show that cultural intelligence conditionally modifies the strength of the indirect relationship between emotional intelligence and creative performance. More specifically, as the level of cultural intelligence increases, the indirect effect of emotional intelligence on creative performance through psychological well-being becomes stronger (B = 0.132, SE = 0.042, 95% CI [0.051, 0.212], *p* < .05). Conversely, at the lower level of cultural intelligence, the conditional indirect association is weaker and its confidence interval includes zero (B = 0.025, SE = 0.038, 95% CI [−0.032, 0.082]). The findings demonstrate that the relationship between emotional intelligence and creative performance, mediated by psychological well-being, can differ by level of cultural intelligence; hypotheses H3 and H4 are also supported.

## 5. Discussion

This study examines the relationship between emotional intelligence and creative performance through psychological well-being and cultural intelligence among employees from two large ICT companies in Turkey. The findings support the basic research model and reveal that emotional intelligence is a significant individual resource for employees’ creative performance. Furthermore, this relationship is not merely a direct effect; psychological well-being and individual capabilities such as cultural intelligence also play a crucial role in this interaction. With this emphasis, the study supports approaches that suggest creativity should be considered not solely in terms of cognitive capacity but also in conjunction with an individual’s psychological functioning and capacity for social adaptation. This result particularly demonstrates the need to move beyond traditional approaches that explain creativity solely in terms of cognitive abilities and technical expertise. Understanding employees’ emotional and psychological resources and how these resources function across different work contexts provides a crucial explanatory framework for creative performance.

The first finding supports H1 by showing a positive association between emotional intelligence and creative performance. This result is consistent with Darvishmotevali et al. (2018), who reported a positive relationship between emotional intelligence and creative performance among frontline hotel employees, and with the meta-analytic findings of Xu et al. (2019). It also complements recent evidence from high-technology firms indicating that the relationship between emotional intelligence and employee creativity may involve additional individual and work-related processes (dos Santos et al., 2025). Given the problem-solving demands, changing task expectations, and team-based work structures of the ICT sector, emotional regulation and interpersonal skills may be relevant to employees’ creative performance alongside their technical knowledge. This suggests that creativity is not merely a cognitive output within a multidimensional structure and interaction mechanism, but also a process intertwined with emotional and social dynamics. Employees in the IT sector, in particular, may face constantly changing customer demands, project-based work structures, and tasks involving a high degree of uncertainty. In software development and technology-oriented business processes, employees are often expected to manage problems that cannot be solved with standard procedures and to adapt quickly to changing conditions (Faraj & Sproull, 2000; Moe et al., 2010). It is believed that employees who can manage their emotional processes more effectively in such work environments can reduce the suppressive effect of negative emotions on cognitive resources, thereby directing their attention and energy toward problem-solving. Indeed, findings suggesting that positive psychological states increase cognitive flexibility and support creativity also support this interpretation (Fredrickson, 2001; Baas et al., 2008).

The findings also support H2, which proposed the mediating role of psychological well-being. The results indicate that psychological well-being accounts for part of the positive association between emotional intelligence and creative performance. This finding is consistent with previous evidence linking emotional intelligence to psychological well-being (Sánchez-Álvarez et al., 2016) and psychological well-being to creativity (Acar et al., 2021). However, these earlier studies largely examined the relevant relationships separately. The present finding brings these relationships together by showing that psychological well-being represents one possible explanatory pathway between emotional intelligence and creative performance. This interpretation is also consistent with the Broaden-and-Build Theory, which suggests that positive psychological experiences broaden individuals’ cognitive and behavioral repertoires (Fredrickson, 2001). Accordingly, employees’ positive psychological functioning may help explain why emotional intelligence is associated with greater creative performance. Given the cross-sectional design, this finding represents an indirect association rather than evidence of a temporal or causal process.

The findings concerning cultural intelligence provide support for the proposed conditional process represented by H3 and H4. The positive association between emotional intelligence and psychological well-being varies across employees’ levels of cultural intelligence, and the related indirect association with creative performance is stronger at higher levels of cultural intelligence. This result is partly consistent with Darvishmotevali et al. (2018), who also identified cultural intelligence as a moderator, although they examined its role in the direct relationship between emotional intelligence and creative performance. The present study differs by locating this moderating role in the emotional intelligence–psychological well-being relationship and the resulting indirect association with creative performance. Cultural intelligence should not, however, be interpreted as a contextual characteristic of the participating organizations. Rather, it represents an individual capability that may help employees understand different social cues, adjust to unfamiliar interaction norms, and use their emotional skills more effectively in culturally diverse situations (Ang et al., 2007; Rockstuhl & Van Dyne, 2018). In this respect, the findings are consistent with the proposed conditional process: emotional intelligence is more strongly associated with psychological well-being among employees with higher cultural intelligence, which is, in turn, associated with stronger creative performance. Thus, cultural intelligence helps clarify why the relationship between employees’ emotional resources, psychological well-being, and creative performance may not be equally strong for all employees.

When the research findings are evaluated as a whole, it becomes clear that employees’ creative performance cannot be explained solely by their technical knowledge, level of expertise, or cognitive competencies. In the information technology sector, employees’ ability to manage emotional processes is especially important for producing creative outputs. However, this effect cannot be evaluated independently of employees’ psychological state and the characteristics of their work environment. In this context, the results show that the effect of emotional intelligence on creative performance is mediated by psychological well-being, and that cultural intelligence can also strengthen this process. Therefore, a more comprehensive explanation of creative performance can be provided by considering employees’ emotional, psychological, and social adjustment-related resources together.

### 5.1. Theoretical Contribution and Managerial Implications

The research makes significant theoretical and practical contributions. The study offers two main theoretical contributions. First, previous research has established a relationship between emotional intelligence and creative performance and has examined environmental uncertainty as an explanatory mechanism (Darvishmotevali et al., 2018). The present findings add to this research by showing that psychological well-being statistically accounts for part of the association between emotional intelligence and creative performance. Psychological well-being is not treated simply as an additional variable; rather, it represents the positive psychological functioning through which employees’ emotional capabilities may be associated with their creative performance. This interpretation is consistent with the Broaden-and-Build Theory, which proposes that positive psychological experiences broaden individuals’ cognitive and behavioral repertoires and support flexible thinking (Fredrickson, 2001; Baas et al., 2008). Given the cross-sectional design, however, the findings should be interpreted as supporting a statistical indirect association rather than demonstrating a causal psychological mechanism.

Second, the findings indicate that this indirect association varies across levels of cultural intelligence. Unlike an organizational or environmental condition, cultural intelligence is conceptualized here as an individual capability that may help employees use their emotional skills across different cultural and interpersonal situations. The study therefore does not claim to extend the Broaden-and-Build Theory. Instead, the results provide context-specific support for its general reasoning by showing that the association between emotional intelligence, positive psychological functioning, and creative performance may differ according to another individual capability.

The findings also offer several practical considerations. Emotional intelligence and psychological well-being were positively associated with creative performance in the participating ICT companies. These results suggest that managers and HR professionals may consider employees’ emotional and psychological resources alongside the technical competencies required for their roles. However, because technical competence was not measured in this study, the findings do not establish that emotional or psychological resources are more important than technical expertise.

Organizations may consider voluntary development activities focusing on emotional awareness, adaptive communication, and intercultural interaction. They may also review work practices that are relevant to psychological well-being, including access to support, team communication, and the management of demanding workloads. These recommendations should be interpreted cautiously, as the cross-sectional findings do not demonstrate that such practices will directly improve creative performance. Rather, they identify areas that may be relevant when designing employee development and well-being initiatives.

### 5.2. Limitations and Future Studies Recommendations

The study has some limitations. First, the research was conducted using a cross-sectional design. Because all variables were measured at the same time, their temporal ordering cannot be established, and reverse relationships or omitted-variable explanations cannot be ruled out. Accordingly, the mediation and moderated mediation findings should be interpreted as conditional indirect associations rather than evidence of causal processes. Future studies using longitudinal designs could provide a clearer understanding of the temporal relationships among the variables. Another limitation of the study is that the data were collected through participants’ self-assessments. This approach may have introduced response bias. Although various measures were taken to reduce common methodological bias during the research process, obtaining the variables from a single data source does not eliminate all risk of bias arising from participant perceptions. Therefore, using managerial evaluations or information from multiple data sources in future studies could yield stronger results. However, it should also be considered that creative performance was measured based on employees’ self-assessments. In future research, considering creative performance alongside managerial evaluations or more objective performance indicators could help provide a more comprehensive evaluation of the findings. In addition, psychological well-being was assessed using a short, three-item measurement tool. While this measure is suitable for assessing the general structure of psychological well-being within the proposed mediation model, it may not fully capture its multidimensional nature, even though it demonstrated sufficient measurement properties in the current sample. Future research should use longer, multidimensional psychological well-being measures and test whether the relationships revealed in this study replicate across specific dimensions of psychological well-being. The sampling procedure and organizational scope also require caution. Participants were recruited on a voluntary basis from two large ICT companies, and random sampling was not used. Therefore, employees who chose to participate may differ from those who did not, creating a possibility of self-selection bias. In addition, company-level differences were not examined separately. Organizational policies, work practices, or climate may be associated with employees’ psychological well-being and creative performance, and these influences cannot be ruled out in the present study. Finally, the inclusion of only two companies from a single country limits the representativeness and generalizability of the findings. The results should therefore be interpreted as applying primarily to the participating employees and organizations. Future studies could examine the model across a larger number of organizations, regions, countries, and sectors and could use probability-based or multilevel sampling designs where feasible.

Relatedly, the sector-specific nature of the study presents a further limitation. Future research could compare the proposed relationships across sectors with different task structures, interaction requirements, and technological intensity. Such comparisons would help determine whether the findings are specific to ICT work or can be observed in other occupational settings. Furthermore, incorporating additional contextual variables, such as leadership style, organizational support, or psychological safety, into the model in future research could help more comprehensively explain how employees’ individual capacities and psychological states affect their creative performance. In addition, future studies examining additional psychological variables beyond psychological well-being, such as self-efficacy, psychological resilience, work engagement, or psychological capital, could contribute to a better understanding of the mechanisms by which emotional intelligence influences creative performance.

## Figures and Tables

**Table 1:** Measurement model.

Items	Factor Loading	AVE	α	CR	ω
Emotional Intelligence		0.597	0.948	0.959	0.950
EI1	0.811				
EI2	0.785				
EI3	0.708				
EI4	0.825				
EI5	0.793				
EI6	0.810				
EI7	0.722				
EI8	0.752				
EI9	0.766				
EI10	0.757				
EI11	0.849				
EI12	0.723				
EI13	0.729				
EI14	0.736				
EI15	0.748				
EI16	0.826				
Psychological Well-Being		0.624	0.824	0.833	0.830
PWB1	0.817				
PWB2	0.769				
PWB3	0.794				
Cultural Intelligence		0.597	0.922	0.930	0.926
CI1	0.835				
CI2	0.827				
CI3	0.744				
CI4	0.765				
CI5	0.829				
CI6	0.733				
CI7	0.717				
CI8	0.726				
CI9	0.768				
Creative Performance		0.555	0.888	0.897	0.895
CP1	0.701				
CP2	0.755				
CP3	0.720				
CP4	0.763				
CP5	0.821				
CP6	0.733				
CP7	0.717				

**Table 2:** Discriminant Validity.

Variable	EI	PWB	CI	CP
Emotional Intelligence (EI)	**(0.772)**	0.297	0.235	0.479
Psychological Well-Being (PWB)	0.222	**(0.790)**	0.378	0.462
Cultural Intelligence (CI)	0.207	0.353	**(0.773)**	0.344
Creative Performance (CP)	0.434	0.410	0.309	**(0.745)**

Footnote. *p* < .05. The diagonal bold values in parentheses represent √AVE values. Values below the diagonal represent interstructural correlation coefficients, while values above the diagonal represent HTMT (Heterotrait–Monotrait) ratios.

**Table 3:** Model Comparisons using a CFA approach.

Variable	χ^2^/df	CFI	GFI	TLI	NFI	RMSEA
Four-Factor (Research Model)	2.489	0.977	0.958	0.949	0.976	0.048
Three-Factor (EI, PWB, CI+CP)	3.967	0.846	0.819	0.821	0.840	0.112
Two-Factor (EI+PWB, CI+CP)	4.122	0.727	0.748	0.704	0.720	0.182
One-Factor (EI+PWB+CI+CP)	5.078	0.641	0.454	0.552	0.637	0.227

**Table 4:** Hypotheses testing.

Path		Coeff	SE	t	*p*	LLCI	ULCI
		Direct Effects					
EI→CP		0.328	0.063	5.205	<.001	0.204	0.450
EI→PWB		0.182	0.032	5.688	<.001	0.121	0.245
PWB→CP		0.287	0.046	6.240	<.001	0.197	0.378
		Indirect effects					
EI→PWB→CP		0.053	0.025			0.009	0.106
		Interaction Effect					
EI*CI → PWB		0.483	0.034	14.206	<.001	0.415	0.551
		Bootstrapping results for conditional indirect effect					
High (+1 SD)		0.132	0.042			0.051	0.212
Medium (M)		0.077	0.035			0.011	0.148
Low (−1 SD)		0.025	0.038			−0.032	0.082
		Moderated mediation					
Index of moderated mediation		0.075	0.030			0.016	0.134

Footnote. * means pwb is affected by the two of these variables together.

## Data Availability

The data presented in this study are available on request from the corresponding author. The data are not publicly available due to privacy, legal or ethical restrictions.
